# Fenofibrate (a PPAR-α Agonist) Administered During Ethanol Withdrawal Reverts Ethanol-Induced Astrogliosis and Restores the Levels of Glutamate Transporter in Ethanol-Administered Adolescent Rats

**DOI:** 10.3389/fphar.2021.653175

**Published:** 2021-04-20

**Authors:** Francisca Villavicencio-Tejo, Osvaldo Flores-Bastías, Lucas Marambio-Ruiz, Diliana Pérez-Reytor, Eduardo Karahanian

**Affiliations:** Institute of Biomedical Sciences, Faculty of Health Sciences, Universidad Autónoma de Chile, Santiago, Chile

**Keywords:** fibrates, alcohol use disorder, alcoholism, treatment, neuroinflammation

## Abstract

High-ethanol intake induces a neuroinflammatory response, which has been proposed as responsible for the maintenance of chronic ethanol consumption. Neuroinflammation decreases glutamate transporter (GLT-1) expression, increasing levels of glutamate that trigger dopamine release at the corticolimbic reward areas, driving long-term drinking behavior. The activation of peroxisome proliferator-activated receptor alpha (PPARα) by fibrates inhibits neuroinflammation, in models other than ethanol consumption. However, the effect of fibrates on ethanol-induced neuroinflammation has not yet been studied. We previously reported that the administration of fenofibrate to ethanol-drinking rats decreased ethanol consumption. Here, we studied whether fenofibrate effects are related to a decrease in ethanol-induced neuroinflammation and to the normalization of the levels of GLT-1. Rats were administered ethanol on alternate days for 4 weeks (2 g/kg/day). After ethanol withdrawal, fenofibrate was administered for 14 days (50 mg/kg/day) and the levels of glial fibrillary acidic protein (GFAP), phosphorylated NF-κB-inhibitory protein (pIκBα) and GLT-1, were quantified in the prefrontal cortex, hippocampus, and hypothalamus. Ethanol treatment increased the levels of GFAP in the hippocampus and hypothalamus, indicating a clear astrocytic activation. Similarly, ethanol increased the levels of pIκBα in the three areas. The administration of fenofibrate decreased the expression of GFAP and pIκBα in the three areas. These results indicate that fenofibrate reverts both astrogliosis and NF-κB activation. Finally, ethanol decreased GLT-1 expression in the prefrontal cortex and hippocampus. Fenofibrate normalized the levels of GLT-1 in both areas, suggesting that its effect in reducing ethanol consumption could be due to the normalization of glutamatergic tone.

## Introduction

The health and social consequences of ethanol use disorder (AUD) have been widely recognized (World Health Organization, 2018). Currently, there are no effective pharmacological therapies to treat AUD since, despite the existence of medications such as naltrexone and acamprosate to reduce the craving for ethanol, most patients relapse in the short or medium-term after initial detoxification (Maisel et al., 2013; Plosker, 2015), suggesting that chronic and abusive consumption of ethanol leaves long-term imprints on the brain, possibly perpetuating the addictive state. This raises the urgent need to find new and better drugs for the treatment of AUD.

Neuroinflammation has been implicated in the consolidation of long-term addiction to ethanol (Coller and Hutchinson, 2012; Flores-Bastías and Karahanian, 2018). The activation of the brain’s innate immune system triggers long-lasting neurobiological changes, e.g., increased glutamate-induced hyperexcitability and excitotoxicity, decreased neurogenesis, and behavioral changes such as anxiety and depression, all of which are related to ethanol abuse (Crews et al., 2011). Although the fact that ethanol intake causes neuroinflammation had been reported (Blanco and Guerri, 2007; Flores-Bastías et al., 2019), the first evidence that neuroinflammation would be a major cause of ethanol addiction came from experiments by Blednov et al. (2011), who reported that the i. p. administration of bacterial lipopolysaccharide (LPS) to mice increased voluntary ethanol intake for a prolonged time. The consumption of ethanol increases the permeability of the intestinal mucosa, enabling the diffusion of bacterial LPS into circulation (Ferrier et al., 2006). LPS entering the body triggers the release of tumor necrosis factor-alpha (TNF-α) to blood, which crosses the blood-brain barrier and activates microglial and astrocytic TNF-α receptors, inducing neuroinflammation through a signaling pathway that leads to NF-κB activation (Qin et al., 2007; Crews and Vetreno 2016). This state of neuroinflammation is maintained for several months (Qin et al., 2007), suggesting the existence of long-lasting mechanisms that perpetuate it. In addition, ethanol upregulates cytochrome P4502E1 (CYP2E1) activity and expression in the brain, and CYP2E1 oxidation of ethanol increases ROS levels which in turn activate NF-κB. Also, NF-κB activation induces the expression of NADPH oxidase (NOX), an enzyme that produces even more ROS, thus creating an activation loop that rapidly induces genes that lead to the activation of the innate immune response (Crews et al., 2011; Flores-Bastías and Karahanian, 2018).

The hippocampus is the brain region most pathologically affected by chronic ethanol ingestion (Franke et al., 1997), which leads to the loss of control over drinking in humans (Crews, 2008; Hermens et al., 2015). The activity of astrocytes and microglia in the hippocampus is exacerbated in alcoholics (He and Crews, 2008). This activation promotes the release of pro-inflammatory cytokines and ROS which in turn, promote neuronal death in the hippocampus and other brain regions (Ward et al., 2009). The sustained activation of neuroinflammation plus the exacerbated production of ROS would lead to neurodegeneration of key areas involved in excessive ethanol intake (Flores-Bastías and Karahanian, 2018). The prefrontal cortex (PFC) is a region that operates within the mesocorticolimbic circuit to exert executive control over ethanol-seeking behavior. Besides the hippocampus, ethanol-induced cell death in other regions such as PFC and the hypothalamus may lead to lack of inhibition of reward areas, reducing behavioral inhibition and increasing motivation to drink (Crews et al., 2011; Crews et al., 2015). Interestingly, it is reported that adolescence is when the greatest damage associated with ethanol-induced neuroinflammation occurs in hippocampal, cortical, and hypothalamic structures, both in humans and in murine models (Crews and Vetreno, 2011; Crews et al., 2016; Pascual et al., 2018). In adolescents, the typical pattern of ethanol consumption corresponds to binge drinking, where a large amount is consumed in a short period time.

The excitatory neurotransmitter glutamate is strongly implicated in the maintenance of chronic ethanol intake, as well as with relapse (Reissner and Kalivas, 2010). Ethanol increases levels of glutamate within corticolimbic structures (Dahchour and De Witte, 2000). Repeated administration of ethanol elicits a progressive increase in the capacity of ethanol to stimulate glutamate release, leading to glutamate sensitization (Szumlinski et al., 2007; Carrara-Nascimento et al., 2011). After chronic ethanol intake and following abstinence, relapse is elicited by cues previously associated with the ethanol effect, which activate glutamate release in several brain areas involved in the memory of the addictive-drug cues. After this memory association, the glutamatergic drive is transferred to PFC, increasing dopamine release at the reward system driving the drinking behavior (Rao et al., 2015; Bell et al., 2016). Glutamate transporter 1 (GLT-1), also known as excitatory amino acid transporter 2 (EAAT2) or solute carrier family 1 member 2 (SLC1A2), is the main transporter that allows re-uptake of glutamate from the extracellular space by astrocytes at tripartite glutamatergic synapses. Glutamate reuptake is imperative to prevent neuronal damage due to exacerbated activity of glutamate receptors. Interestingly, animals that consume ethanol chronically show reduced levels of GLT-1 and marked increases in extracellular levels of glutamate (Sari et al., 2013a). The administration of ceftriaxone (a drug that increases the levels of GLT-1) to ethanol-drinking rats restored extracellular glutamate concentration and reduced chronic ethanol intake by 70% (Sari et al., 2013b; Das et al., 2015). In addition to ethanol consumption, neuroinflammation induced by direct administration of LPS or TNF-α also reduces the levels of GLT-1, leading to an increase in extracellular glutamate; blockade of the signaling produced by proinflammatory cytokines normalized GLT-1 and levels of glutamate (Fang et al., 2012). Thus, it is conceivable that the reduction in GLT-1 expression produced by ethanol is mediated by the activation of the neuroinflammatory response.

Peroxisome proliferator-activated receptor alpha (PPAR-α) is a nuclear receptor that has essential functions in the metabolism of lipids (Berger and Moller, 2002). PPAR-α can be activated by fibrate drugs, which include fenofibrate, bezafibrate, gemfibrozil, ciprofibrate, and clofibrate (Schoonjans et al., 1996). Fibrates increase the oxidation rate of fatty acids; therefore, they are widely used clinically for the treatment of hypertriglyceridemia (Cignarella et al., 2006). Several authors have reported that fenofibrate decreases ethanol intake in rodents (Barson et al., 2009; Karahanian et al., 2014; Blednov et al., 2015, 2016; Haile and Kosten, 2017; Rivera-Meza et al., 2017). We were able to demonstrate that fenofibrate increases catalase and alcohol dehydrogenase activities in the liver, which leads to rapid acetaldehyde accumulation when the animals drink ethanol (Karahanian et al., 2014; Rivera-Meza et al., 2017; Muñoz et al., 2020). Acetaldehyde that accumulates in the blood produces unpleasant effects that lead to an aversion to ethanol consumption in animal models and humans (Mizoi et al., 1979). Additionally, fenofibrate would have effects at the central level that decrease the craving to drink ethanol after a period of abstinence (Rivera-Meza et al., 2017). Interestingly, astrocytes are the cell type where PPARα is most expressed in the brain (Cullingford et al., 1998). This opens the possibility that the activation of PPARα in astrocytes by fenofibrate could be mediating its effects at the central level. Given the central role of astrocytes in the neuroimmune system, we hypothesized that fenofibrate should have a modulating effect on neuroinflammation induced by ethanol consumption.

To date, the effect of the activation of PPAR-α on ethanol-induced neuroinflammation has not been reported. However, the decrease in neuroinflammation mediated by PPAR-α activation has been reported in brain injury models other than ethanol abuse (aging, ischemia/reperfusion injury, and traumatic brain injury) (Poynter and Daynes, 1998; Bordet et al., 2006; Collino et al., 2006; Chen et al., 2007; Shehata et al., 2020). Activation of PPAR-α by the administration of fibrates downregulates NF-κB activity (Staels et al., 1998; Delerive et al., 1999, 2000; Marx et al., 1999; Collino et al., 2006), decreasing the production of proinflammatory cytokines and enzymes (Poynter and Daynes, 1998; Pahan et al., 2002; Bordet et al., 2006; Chen et al., 2007).

Here, we aimed to evaluate whether fenofibrate administered during ethanol withdrawal would be capable of reducing neuroinflammation induced by binge ethanol ingestion in adolescent rats and of restoring the levels of GLT-1 in the hippocampus, hypothalamus, and prefrontal cortex, which could help to explain the protracted effects of this drug on reducing the craving to drink ethanol after periods of abstinence. To evaluate the anti-neuroinflammatory effects of fenofibrate after a prolonged period of administration of ethanol, we quantified by western blot the levels of the neuroinflammation marker proteins GFAP and inhibitor of κB alpha (IκBα) phosphorylated. GFAP increases its expression when astrocytes become activated in neuroinflammatory processes. On the other hand, IκBα is the protein that binds to and inhibits NF-κB, masking its nuclear localization signal which keeps it sequestered in the cytoplasm. When inflammatory processes are triggered, IκBα is phosphorylated (pIκBα), which causes it to become detached from NF-κB, which is now free to enter the nucleus where it can activate the expression of specific genes related to the immune response.

## Method

Male Sprague-Dawley rats were purchased from Pontificia Universidad Católica de Chile. Rats were housed in individual cages in a temperature-controlled room on a 12-h light/12-h dark cycle, with food and water provided *ad libitum*. The binge-like administration of ethanol was started by esophageal gavage in 30-day-old adolescent rats (*n* = 4 per group). Administration of ethanol was carried out for 4 weeks on Mondays, Wednesdays, and Fridays, starting the first week with a dose of 1 g/k/day ethanol and continuing the following 3 weeks with a dose of 2 g/k/day (both doses as a 30% v/v ethanol solution). Control groups were intragastrical given only water (*n* = 4 per group). At the end of 4 weeks, the administration of ethanol was terminated, and ethanol-treated animals and controls were randomly separated into two groups each. One ethanol group and one control group were administered micronized fenofibrate 50 mg/k/day (Fibronil, Royal Pharma, Chile) by esophageal gavage for 14 days. The other two groups were given only vehicle.

One day after the last dose of fenofibrate or vehicle, the animals were anesthetized with ketamine/xylazine (10:1 mg/kg of body weight, i. p.), decapitated and the brains removed. *Hippocampus*, hypothalamus, and prefrontal cortex tissues were extracted and homogenized with an Ultra-Turrax homogenizer in buffer containing Tris-HCl 100 mM pH 7.4, EDTA 5 mM, SDS 1%, PMSF 1 μM and a protease inhibitor cocktail (Pierce) (1 ml lysis buffer per 0.1 g tissue). Then, the samples were centrifuged at 14.000 x g for 10 min at 4 °C to remove tissue debris. Protein concentration was determined in the supernatants with the Bio-Rad protein assay kit. SDS-PAGE was performed loading the same amount of total protein per lane (100 μg). The expression levels of GLT-1, pIκBα and GFAP were determined by western blot with the following antibodies: anti-GLT-1 (PA5-19706 ThermoFisher Scientific), anti-pIκBα (SC8404, Santa Cruz Biotechnology, detects specifically IκBα phosphorylated at Ser 32), and anti-GFAP (G3893 Sigma-Aldrich). As a loading control, the levels of *ß*-actin were also determined in the same blots (anti-β-actin SC47778, Santa Cruz Biotechnology). All primary antibodies were used at 1:3,000 dilution, and were incubated at 4 °C overnight, except for *ß*-actin and GLT-1, which were incubated for 1:30 h at room temperature. After the incubation with the corresponding HRP-conjugated secondary antibodies (diluted 1:5,000), blotting membranes were revealed with Luminata Western HRP Chemiluminescence Substrate (Merck Millipore). The different proteins and *ß*-actin were all quantified in the same blotting membranes, which were previously stripped of the preceding antibodies by incubation for 10 min in Restore Western Blot Stripping Buffer (ThermoFisher Scientific) (the detection sequence was: pIκBα, GFAP, GLT-1 and finally *ß*-actin). Bands were quantified by densitometry with ImageJ software. The levels of each protein were expressed as percentages of their water-administered controls, normalized by the levels of *ß*-actin. For statistical analysis, two-way ANOVA was used followed by Bonferroni's post-hoc analysis.

## Results

**FIGURE 1:**
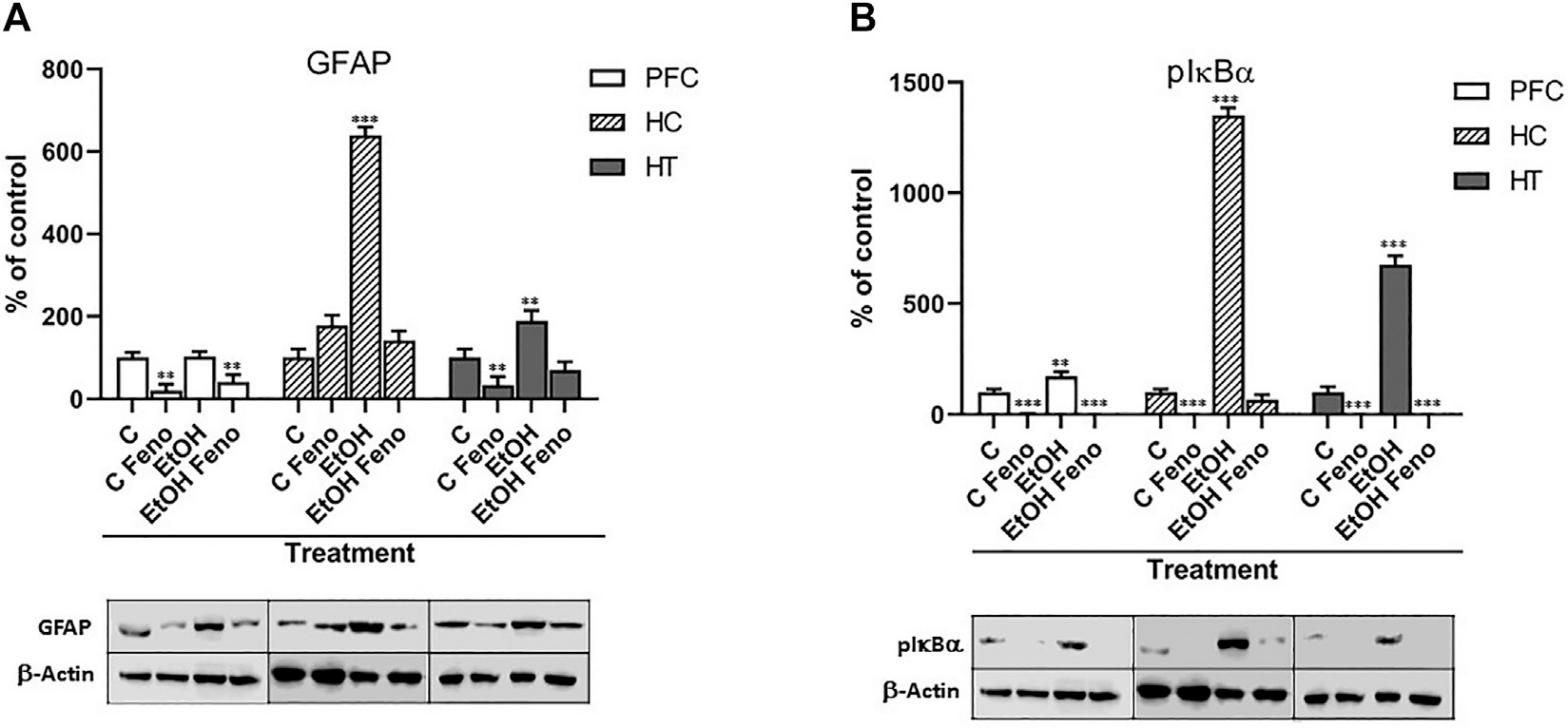
Expression levels of GFAP **(A)** and pIκBα **(B)** in prefrontal cortex (PFC), hippocampus (HC) and hypothalamus (HT) in rats that were administered alcohol for one month and then were treated with fenofibrate for 14 days. C = water-administered controls; C Feno = water-administered controls treated with fenofibrate; EtOH = alcohol-treated rats; EtOH Feno = alcohol-treated rats then treated with fenofibrate. The graphs show the protein levels expressed as percentages of their water-administered controls, normalized by the levels of *ß*-actin. Representative blots from each group are shown below each respective bar. ANOVA **p < 0.01; ***p < 0.001. GFAP PFC: F_(3,12)_ = 23.50 for Feno effect; GFAP HC: F_(3,12)_ = 131.10 for EtOH effect; GFAP HT: F_(3,12)_ = 34.07 for EtOH effect, F_(3,12)_ = 75.40 for Feno effect. pIκBα PFC: F_(3,12)_ = 9.76 for EtOH effect, F_(3,12)_ = 149.90 for Feno effect; pIκBα HC: F_(3,12)_ = 1,190.00 for EtOH effect, F_(3,12)_ = 1,314.00 for Feno effect; pIκBα HT: F_(3,12)_ = 594.40 for EtOH effect, F_(3,12)_ = 1,080.00 for Feno effect. The error bars correspond to SD, *n* = 4. The absolute values, normalized by *ß*-actin, obtained from the quantification of the bands are **(A)** GFAP: PFC (C = 0.98 ± 0.33; C FENO = 0.20 ± 0.08; EtOH = 1.00 ± 0.13; EtOH FENO = 0.40 ± 0.09); HC (C = 0.31 ± 0.11; C FENO = 0.55 ± 0.03; EtOH = 1.98 ± 0.04; EtOH FENO = 0.44 ± 0.03); HT (C = 0.56 ± 0.19; C FENO = 0.19 ± 0.05; EtOH = 1.06 ± 0.08; EtOH FENO = 0.39 ± 0.05) **(B)** pIκBα: PFC (C = 0.35 ± 0.12; C FENO = 0.00 ± 0.00; EtOH = 0.60 ± 0.05; EtOH FENO = 0.00 ± 0.00); HC (C = 0.12 ± 0.04; C FENO = 0.00 ± 0.00; EtOH = 1.62 ± 0.02; EtOH FENO = 0.08 ± 0.01); HT (C = 0.16 ± 0.06; C FENO = 0.00 ± 0.00; EtOH = 1.08 ± 0.02; EtOH FENO = 0.00 ± 0.00).

One-month ethanol treatment increased the levels of GFAP in the hippocampus and hypothalamus (to 640 ± 14% and 190 ± 13% of their controls without ethanol, respectively), indicating a clear astrocytic activation in these areas of the brain (Figure 1A). In contrast, the levels of GFAP were not altered by ethanol in the PFC. It should be noted that these effects were measured 14 days after finishing the administration of ethanol. The administration of fenofibrate for 14 days to rats that were previously treated with ethanol markedly decreased the expression of GFAP in the PFC, hippocampus, and hypothalamus (to 40 ± 7, 141 ± 9 and 69 ± 7% of their controls without ethanol, respectively), which indicates that fenofibrate reverses ethanol-induced glial activation. Interestingly, in PFC and hypothalamus, fenofibrate was able to reduce the expression levels of GFAP even in rats that had not been treated with ethanol. In the hippocampus, there appears to be a slight increase in GFAP expression in rats that were treated with fenofibrate, however, this is not statistically significant.

The levels of pIκBα increased in the PFC, hippocampus, and hypothalamus due to the administration of ethanol (to 170 ± 11, 1,350 ± 14 and 675 ± 13% of their controls without ethanol, respectively) (Figure 1B). Again, it is important to note that this effect was measured 14 days after finishing the administration of ethanol. Administration of fenofibrate to rats that had previously been treated with ethanol decreased the inactivation of IκBα to levels even lower than its controls without ethanol, in the three brain areas. Furthermore, fenofibrate reduced the levels of pIκBα to undetectable values in control animals, as well as in the PFC and hypothalamus of ethanol-treated rats. These results indicate that ethanol treatment induces phosphorylation of IκBα, which would allow the activation of NF-κB, triggering the neuroinflammatory response. Fenofibrate markedly reverts this effect, again showing its ability to reverse neuroinflammation induced by the administration of ethanol.

**FIGURE 2:**
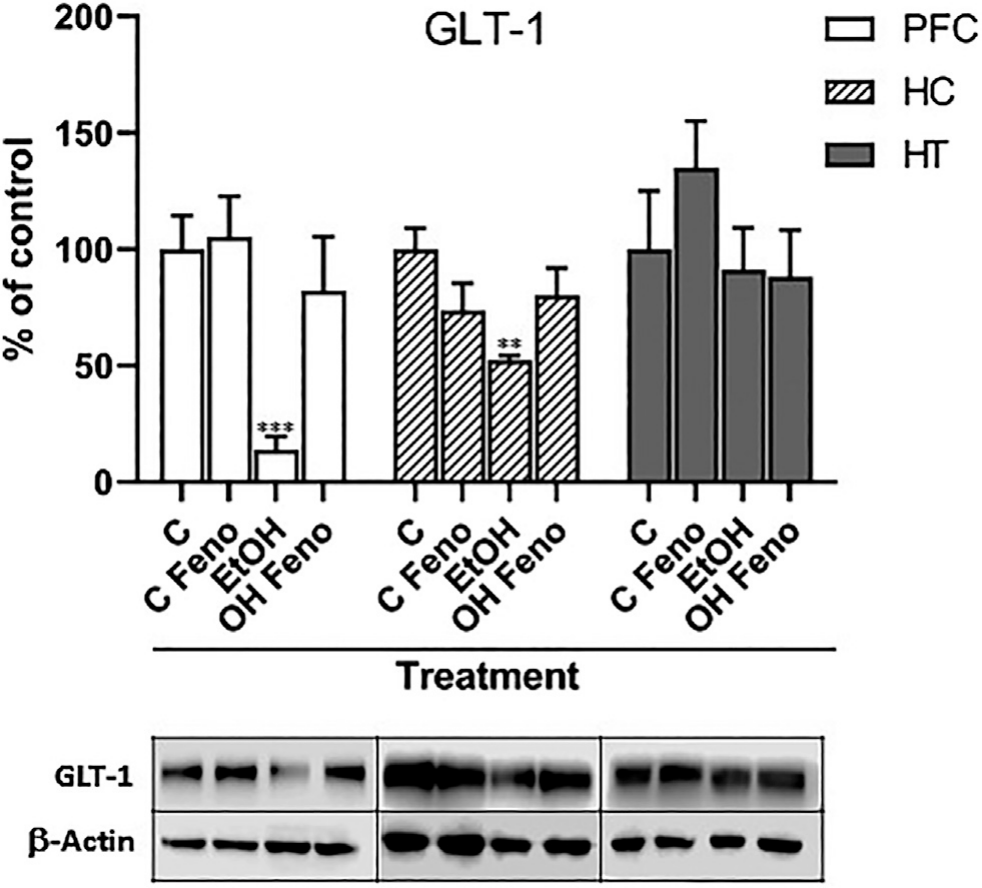
Expression levels of GLT-1 in prefrontal cortex (PFC), hippocampus (HC) and hypothalamus (HT) in rats that were administered alcohol for one month and then were treated with fenofibrate for 14 days. C = water-administered controls; C Feno = water-administered controls treated with fenofibrate; EtOH = alcohol-treated rats; EtOH Feno = alcohol-treated rats then treated with fenofibrate. The graphs show the protein levels expressed as percentages of their water-administered controls, normalized by the levels of *ß*-actin. Representative blots from each group are shown below each respective bar. ANOVA **p < 0.01; ***p < 0.001. PFC: F_(3,12)_ = 14.81 for EtOH effect; HC: F_(3,12)_ = 6.39 for EtOH effect. The error bars correspond to SD, *n* = 4. The absolute values, normalized by *ß*-actin, obtained from the quantification of the bands are: PFC (C = 1.30 ± 0.44; C FENO = 1.37 ± 0.11; EtOH = 0.18 ± 0.10; EtOH FENO = 1.07 ± 0.12); HC (C = 0.61 ± 0.21; C FENO = 0.45 ± 0.05; EtOH = 0.32 ± 0.08; EtOH FENO = 0.49 ± 0.06); HT (C = 0.34 ± 0.12; C FENO = 0.51 ± 0.03; EtOH = 0.31 ± 0.05; EtOH FENO = 0.30 ± 0.03).

Administration of ethanol decreased the levels of the glutamate transporter GLT-1, both in the PFC and in the hippocampus (to 14 ± 7% and 52 ± 12% of their controls without ethanol, respectively), but not in the hypothalamus (Figure 2). The administration of fenofibrate for 14 days to rats that had previously been treated with ethanol normalized the expression of GLT-1 in both the PFC and hippocampus (to 82 ± 9 and 80 ± 9% of their controls without ethanol, respectively), which indicates that fenofibrate reverses the ethanol-induced decrease in the levels of GLT-1 which could be leading to the normalization of glutamatergic tone increased by excessive ethanol intake.

## Discussion

The main findings in this study are that the administration of fenofibrate can reverse in the PFC, hippocampus, and hypothalamus, both neuroinflammation and the decrease in the expression of GLT-1, induced by high binge-like administration of ethanol to adolescent rats. Since neuroinflammation has been strongly implicated in the activation of brain mechanisms that perpetuate chronic ethanol consumption, and since fenofibrate is a drug approved for many years by the FDA for the clinical treatment of hypertriglyceridemia, our findings could have a great translational value for the treatment of AUD.

The hippocampus is the brain region that suffers the greatest damage due to neuroinflammation induced by ethanol consumption (Franke et al., 1997; He and Crews, 2008). The exacerbated activation of the glia finally leads to neuronal death in this area (Ward et al., 2009). In agreement with this, we found in our model that the hippocampus is where the greatest increase (640 ± 14%) in the expression of GFAP is detected because of the administration of ethanol (Figure 1A). Besides, we also observed a significant increase in the levels of GFAP in the hypothalamus (190 ± 13%), which agrees with what was observed in the brains of alcoholics where a greater number of GFAP-positive astrocytes exist in hypothalamic tissue compared to non-alcoholic individuals (Cullen and Halliday, 1994). Administration of fenofibrate to rats that had been treated with ethanol reduced GFAP expression to similar levels as their non-ethanol controls, demonstrating that fenofibrate is capable of completely reversing ethanol-induced glial activation. On the other hand, we did not observe changes in the levels of GFAP in PFC of rats treated with ethanol. Interestingly, even though ethanol did not increase GFAP expression in PFC, fenofibrate was still able to reduce the levels of GFAP in control animals and in those that were treated with ethanol (to 20 ± 8 and 40 ± 9% of the controls without ethanol, respectively). A similar effect was observed in the hypothalamus, where fenofibrate also reduced GFAP expression in control animals (to 34 ± 8% of their controls without fenofibrate).

We also evaluated the ability of fenofibrate to inhibit ethanol-induced phosphorylation of IκBα, and thus inhibit NF-κB activation and neuroinflammatory response. As with GFAP, we observed that the administration of ethanol produced the most marked effects in the hippocampus (increase in pIκBα to 1,350 ± 14% compared to its controls without ethanol), followed by the hypothalamus (675 ± 13%) and PFC (170 ± 11%). This would indicate that the neuroinflammatory response is exacerbated mainly in the hippocampus, which agrees with the fact that this brain region is the one that suffers the greatest damage because excessive ethanol consumption. In all three areas, the administration of fenofibrate reduced the levels of pIκBα to undetectable values, which demonstrates its effectiveness in reducing neuroinflammation induced by the administration of ethanol. Even in non-ethanol control rats, fenofibrate showed effects by completely inhibiting IκBα phosphorylation. PPAR-α can act as a negative regulator of several pro-inflammatory genes via inhibiting the activation of NF-κB (Delerive et al., 2000; Kleemann et al., 2003; Gervois et al., 2004). Activation of NF-κB is initiated by the proinflammatory signal-induced phosphorylation of IκB. This occurs primarily via activation of a kinase called IκB kinase (IKK). Okayasu et al. 2008 demonstrated that TNF-α stimulates IκB phosphorylation by inducing IKK activity, and fenofibrate inhibits TNFα-induced IKK activity and IκB phosphorylation. However, the direct mechanism by which fenofibrate inhibits IKK has not yet been found; apparently, there could be an indirect mechanism mediated by the activation of AMP-activated protein kinase (AMPK), which in fact would phosphorylate IKK, inactivating it (Okayasu et al., 2008).

It is worth analyzing the notorious increase in pIκBα levels produced by ethanol administration, especially in the hippocampus and hypothalamus. Once proinflammatory signals are present due to ethanol consumption, NF-κB is activated and commands the expression of its own repressor, IκBα. The newly synthesized IκBα then re-inhibits NF-κB and, thus, forms a feedback loop of regulation (Nelson et al., 2004). Thereby, as long as NF-κB is active the levels of IκBα increase, which in turn will be phosphorylated by IKK that is active due to the proinflammatory signals present due to ethanol consumption. Thus, the more active NF-κB is maintained, the higher the levels of phosphorylated IκBα are present.

In this study, PFC was the area that showed the least neuroinflammatory response due to alcohol administration, evidenced by the non-increase in the levels of GFAP and a lower phosphorylation response of IκBα (compared to the hippocampus and hypothalamus). It should be emphasized that the measurements were made 2 weeks after finishing with the administration of ethanol; Bull et al. 2015 reported that after 3 weeks of ethanol abstinence, the number of GFAP-positive astrocytes in PFC decreased to the same levels as in control rats that had not consumed ethanol. Similarly, it is conceivable in our studies that 2 weeks of abstinence would have been enough for GFAP expression to normalize in the PFC of rats that had been treated with ethanol. Another possibility is that PFC has a lower capacity to induce an inflammatory response against exposure to ethanol, or that it has a greater anti-inflammatory and/or antioxidant capacity against the injuries produced by ethanol. Consistent with this idea, Kane et al. (2014) reported that ethanol did not increase the expression of pro-inflammatory molecules such as CCL2, IL-6, or TNF-α in adolescent mice cerebral cortex.

As indicated above, ethanol intake increases the levels of glutamate within corticomesolimbic structures, mainly PFC, hippocampus, and nucleus accumbens (Dahchour and De Witte, 2000). This hyper glutamatergic state is responsible for craving in individuals addicted to ethanol and can trigger the neurobiological mechanisms responsible for relapse after a period of abstinence. These mechanisms are related to the memory of ethanol-related cues, which once presented in this hyper glutamatergic context in the hippocampus, the drive is exacerbated transmitted to the PFC, enhancing the behavior of ethanol consumption (Bell et al., 2016). As indicated above, the main cause of this hyper glutamatergic state is the decreased expression of GLT-1. In accordance with what was reported by other research groups (Sari et al., 2013a; Israel et al., 2021), in our studies we observed that ethanol greatly decreased the levels of GLT-1 in PFC and the hippocampus. Interestingly, the administration of fenofibrate reversed this decrease in both areas. On the contrary, in the hypothalamus, we did not observe statistically significant changes in the expression of GLT-1 due to the administration of ethanol or treatment with fenofibrate. To our level of knowledge, alterations of the levels of GLT-1 in the hypothalamus due to the administration of ethanol have not yet been reported. Interestingly, multiple PPAR response elements (PPREs) in the promoter region of the GLT-1 (EAAT2) gene have been found (Romera et al., 2007), suggesting that the activation of PPAR-α by fenofibrate would have a direct effect on the increase of the expression of this gene.

Although GLT-1 is responsible for >90% of the glutamate transport activity in the brain (Holmseth et al., 2009), there are other glutamate transporters, such as the cystine-glutamate antiporter (xC^−^), that participate in the homeostasis of this neurotransmitter. xC^−^ promotes the release of glutamate to the extracellular space while cystine is taken up by the cell. Although its importance in maintaining the levels of glutamate in the synaptic space may not be as relevant as that of GLT-1, the uptake of cystine by glial cells promotes the synthesis of glutathione (of which cystine is its precursor), which is one of the main antioxidant agents that counteract oxidative stress in the brain (Danbolt, 2001). Thus, we cannot rule out that, at least in part, the anti-inflammatory effects of fenofibrate may be due to an upregulation of xC^−^ and an increased synthesis of glutathione.

Although our studies suggest that the decrease in neuroinflammation together with the normalization of the levels of GLT-1 could be responsible for the effects of fenofibrate on alcohol consumption at the central level, we cannot rule out that there are other mechanisms involved. In a previous study (Rivera-Meza et al., 2017), we reported that fenofibrate is also capable of reducing the consumption of another reinforcing substance (saccharin) that a priori would not be related to alcohol; however, there is evidence that ethanol and palatable foods (e.g., sweet taste) ingestion share common mechanisms involving *µ*-opioid receptors and dopaminergic transmission in the brain reward system (Di Chiara, 1998; Kelley et al., 2002; Hajnal et al., 2004). Therefore, this effect of fenofibrate on saccharin consumption could probably be related to a decrease in the dopaminergic response in the limbic reward system. It has been reported that the activation of PPARα induces some tyrosine kinase(s) which has not yet been identified, which phosphorylates and negatively regulates α4β2-type nicotinic acetylcholine receptors, thus decreasing the dopaminergic activity of neurons in the ventral tegmental area (Melis et al., 2008; Melis et al., 2013). These results suggest that fenofibrate-mediated activation of PPARα would also diminish dopamine release in the mesolimbic pathway, thereby decreasing its rewarding activity. We intend to address this hypothesis in future studies.

Overall, our studies show that the administration of fenofibrate to rats that were administered ethanol reverses: (i) the increase in the expression of an astrocyte-activation marker protein (GFAP) in the hippocampus and hypothalamus, (ii) the increase in the levels of phosphorylation of the inhibitory protein of NF-κB (IκB) in PFC, hippocampus, and hypothalamus, which would prevent the activation of neuroinflammation-related genes, and (iii) the decrease in the expression of the glutamate transporter GLT-1, which could possibly lead to the restoration of normal glutamatergic tone, which was altered by ethanol consumption. The latter would lead to a decrease in ethanol craving, which is the main cause of relapse in patients who have detoxified. These findings suggest that the administration of fenofibrate in the withdrawal period after ethanol detoxification could be a good therapeutic alternative to avoid or reduce the severity of relapse.

## Data Availability

The original contributions presented in the study are included in the article/Supplementary Material, further inquiries can be directed to the corresponding author.
